# Case report: Utilization and efficacy of large-bore catheters in mechanical thrombectomies

**DOI:** 10.3389/fneur.2022.1035959

**Published:** 2023-01-10

**Authors:** Meghna Bhattacharyya, Clint A. Badger, Brian T. Jankowitz, Hamza A. Shaikh

**Affiliations:** ^1^Department of Neurosurgery, Cooper University Hospital, Camden, NJ, United States; ^2^Department of Radiology, Cooper University Hospital, Camden, NJ, United States

**Keywords:** mechanical thrombectomy, large-bore catheters, safety, efficacy, stroke

## Abstract

Thrombotic strokes are caused by occlusion of flow in a blood vessel by a clot or thrombus, resulting in disruption of oxygen and nutrients to the brain that can result in neurological deficits. There are many devices now available for safe and effective removal of thrombi from large blood vessels. This report focuses on the Zoom 0.088” large-bore catheter, which has the potential to be navigated into a large vessel for thrombus removal *via* aspiration, and weigh the risks and benefits of its utilization in thrombectomy patients. In this case, we discuss the use of this device for thrombectomy of a left M1 middle cerebral artery occlusion that resulted in a distal left MCA dissection and eventual loss of access to the site of the thrombus. Ultimately, the patient died from a large stroke in the left MCA territory. In light of this occurrence, we seek to explore the utility and feasibility of large-bore catheters and their risks in thrombectomy candidates.

## Background

Thrombotic strokes account for approximately one-half to two-thirds of strokes across the U.S. Commonly seen in the elderly population, notable risk factors include severe atherosclerosis and cardiac arrhythmias, such as atrial fibrillation, which predisposes patients to formation of thrombi that break off and travel into small vessels. While blood flow can be salvaged with the administration of Tenecteplase and other thrombolytic agents, they can only be done so within a 4.5-h time span from the patient's last known normal time. Mechanical thrombectomy, however, offers another opportunity for clot removal within a longer 24-h period and has been proven to be safe and efficacious for patients in which thrombolytic therapy is contraindicated (1).

According to the current literature, mechanical thrombectomy is safe in patients with intracranial large-artery occlusions and by employing a standardized rescue therapy (i.e., stenting and/or angioplasty), patients could benefit from this procedure (2). In fact, mechanical thrombectomy with stent retrievers has been shown to be effective in acute ischemic stroke of the anterior circulation caused by intracranial large artery occlusion (ILAO) in several randomized controlled trials (2). However, it should be noted that the techniques used during catheterization and clot mechanics can play a major role in the outcome as there is the possibility of clot reformation (3).

As of more recently, there have been multiple reports and studies done supporting the use of larger catheters during thrombectomies. In the majority of cases, thrombus aspiration resulted in successful recanalization after a short procedure time. With the additional use of stent retrievers, a high recanalization rate can be achieved (96.5%) (4). Moreover, catheter selection is not an independent predictor of a successful first-pass, final reperfusion, or clinical outcome (5).

The Zoom 0.088”catheter has shown to be quite effective in thrombus retrieval as it gets as close to the blockage as possible. The beveled tip allows for better suction force and there is smooth 1:1 advancement, offering a more supportive access platform. Essentially, intracranial navigation of 0.088”large-bore catheters in mechanical thrombectomies appears technically feasible and safe (6). This catheter itself comes in four sizes (0.071”, 0.055”, 0.045”, and 0.035”Internal Diameter) and is designed to enable seamless tracking through challenging vasculature. Coupled with access catheters, navigating the brain's highly complex and tortuous vascular system becomes a much less daunting task.

At Cooper University Hospital, strokes are treated based on a clinical finding of a disabling deficit along with an ASPECT score > 7 and visually seen blockage on CTA of the head. This institution uses aspiration thrombectomy as its first line with large-bore catheters. In this case, the patient had a left M1 MCA occlusion along with an ASPECT score of 10 and a disabling stroke with an initial NIHSS of 17. The procedure was initiated with an 8-French sheath placed in the right groin. Through that, a Zoom 088/Simmons-2 construct was utilized to access the left common carotid artery. After this, the Simmons-2 was removed and a Zoom 71/velocity microcatheter construct was navigated into the left M1 MCA. The Zoom 088 was then navigated over the Zoom 71 into the left M1 MCA. However, the Zoom 088 inadvertently overshot the thrombus while removing the Zoom 71.

Pure manual aspiration thrombectomy (MAT) was performed resulting in recanalization of the ICA with the M1 occlusion. However, post treatment angiography demonstrated a non-flow limiting dissection of the left M1 MCA.

How effective and efficient large-bore catheters can be exactly must be studied further as methods for improvement continue to be tested with trial and error. While reperfusion catheter failures resulting in injury or death are rare and FDA surveillance of any outliers has proven effective, here, we present a case of an unfavorable post-operative outcome after use of a 0.088”Zoom catheter (7).

## Case presentation

**Figure 1:**
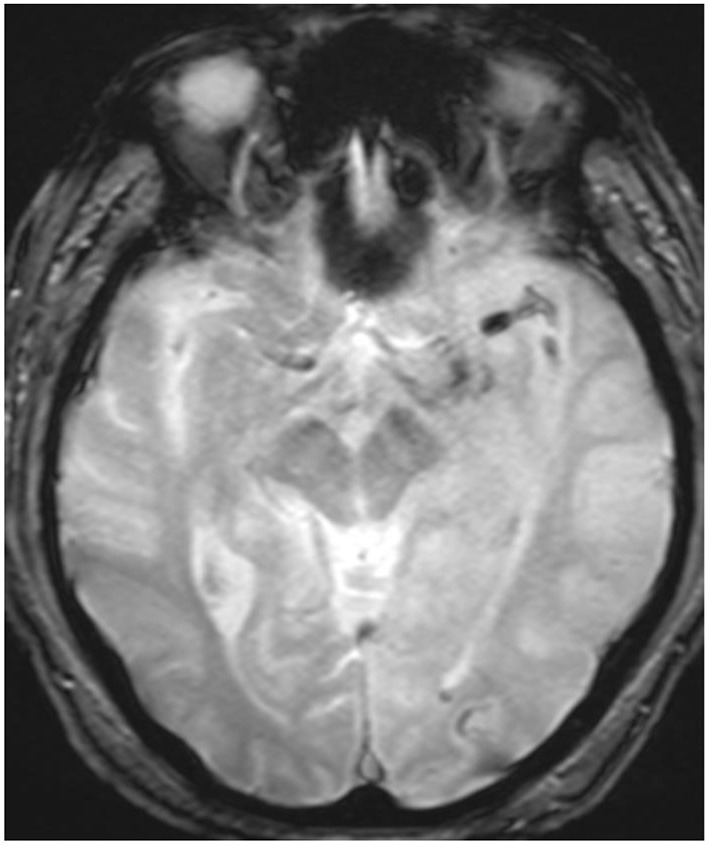
Initial hemoflash image demonstrating a thrombus in the left M1 branch of the left MCA prior to MAT.

**Figure 2:**
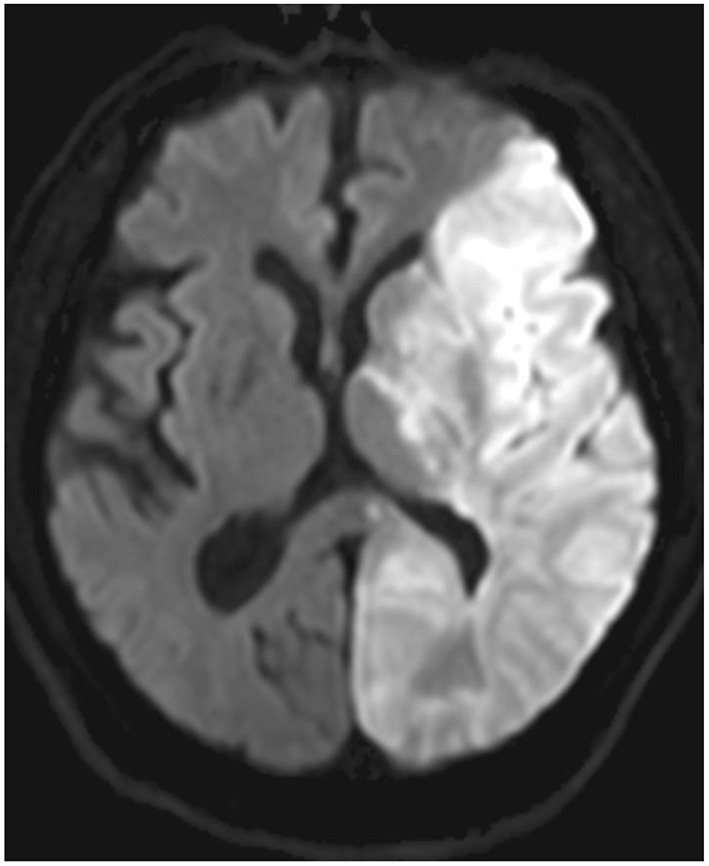
Diffusion-weighted imaging demonstrating the topographical distribution of the left MCA stroke.

**Figure 3:**
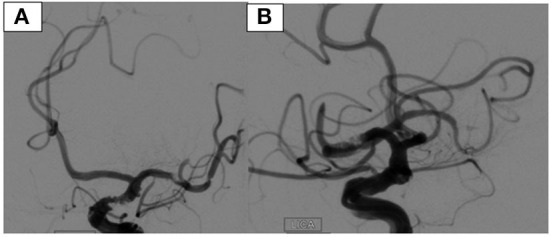
AP **(A)** and lateral **(B)** views of a left ICA dissection after MAT was performed.

A 71-year-old male with a past medical history significant for alcohol abuse presented to the emergency department with right hemiparesis and aphasia from an outside hospital. He was performing his usual activities of daily living when he slumped over. Upon admission, he was not following commands and was found to be in atrial fibrillation with hemoflash imaging indicative of a left M1 hyperdensity, as shown in Figure 1, and diffusion-weighted imaging demonstrating the extent of the stroke in Figure 2. He had an ASPECT score of 10 and an NIHSS of 17. The patient was subsequently administered 0.25 mg/kg of Tenecteplase, approximately 1.5 h after his last known normal time, at which point his NIHSS increased to 19. He was unable to protect his airway and was intubated, after which he was taken directly to the catheterization lab for a left ICA thrombectomy, TICI 0 to 2B. While the immediate outcome after the operation was uncomplicated, the catheter was inadvertently overshot past the thrombus, which resulted in an M1 MCA dissection during the procedure, pictured in Figures 3A, B.

### Treatment

After informed consent the patient was brought into the angiography suite and placed supine on the angiographic table. The right groin was prepped and draped using sterile techniques. The skin overlying the right femoral artery was locally anesthetized with 1% Lidocaine. Using Standard Micropuncture with fluoroscopic guidance, the right femoral artery was accessed, a small skin incision was made, and an 8-French 65-cm sheath was placed. A Zoom 088 was advanced over a Zoom 71 over a velocity microcatheter into the left ICA occlusion. Pure MAT was performed resulting in recanalization of the ICA with continued M1 occlusion. Next, the Zoom 71/velocity construct was navigated into the left M1 MCA and the Zoom 088 was then navigated over the Zoom 71 into the left M1 thrombus. The Zoom 71/velocity construct was removed, at which point the Zoom 088 moved further past the clot into the distal M1/proximal M2 segment interface. Repeat manual aspiration with the Solitaire 4 × 40 stent retriever was performed for further clot retrieval, resulting in TICI 2B flow with a grade 2 dissection of the M1 MCA division. Multiple attempts were made to re-catheterize the MCA, but were unsuccessful due to progressive narrowing from the dissection. At the completion of the procedure, the catheter and sheath were removed and hemostasis in the right groin was obtained by placement of an 8-French Angioseal arteriotomy closure device. The device was deployed without complication. No new neurological deficits or complications were encountered during or immediately following the procedure. The patient was then transferred to the neurological ICU intubated and sedated.

## Outcome and follow-up

After it was found the patient had a dissection, neurosurgery discussed the possibility of a decompressive hemicraniectomy with the patient's wife due to significant swelling on imaging. She declined at the time due to the patient's poor prognosis. On repeat MRI, there was a large left hemispheric infarct within the vascular distributions of the middle and posterior cerebral arteries with no evidence of parenchymal hemorrhage. Additionally, a left-to-right midline shift of approximately 4.5 mm on post-operative day one was appreciated. An intra-arterial thrombus within the M1 branch of the left middle cerebral artery and M2 branches was found. The patient remained intubated due to neurological deficits and lack of improvement of mental status. He was ventilator-dependent until he was transitioned to comfort care and was palliatively extubated on post-operative day two. The patient passed on post-operative day three.

## Discussion

Traditionally, the treatment of intracranial occlusions involves medical therapy in the form of thrombolytics. However, not every patient is a candidate for the usual standard of treatment and first-line treatment may not always be successful. Moreover, if the patient's last known normal time exceeds more than 4.5 h, they are generally just given aspirin and managed medically.

As an alternative, mechanical thrombectomies have shown to be quite successful. For the procedure to show promising results, neurointerventional surgeons lean heavily on their equipment, i.e., the use of catheters. In general, these catheters have a proximal outer diameter of approximately 0.08 inches and a proximal inner diameter of approximately 0.07 inches. They are advanced into the vascular system using the Seldinger technique, which is modified accordingly depending on the target site of interest. In this case, the Zoom 0.088”catheter was utilized. It is the first stroke-specific 0.088”access catheter designed to get closer to the clot. Consistently reaching intracranial anatomy, it minimizes the distance to retrieve the clot, while providing flow control with an outer diameter closely matched to the vessel size. Maximizing the catheter-to-vessel size facilitates near flow-arrest on catheter insertion, potentially negating the need for a balloon-guide catheter. For instance, a 0.088 inch aspiration catheter enables significant flow reversal in the distal MCA during aspiration (8). According to some recent studies, catheters with an inner diameter of 0.040 inch and 0.064 inch, respectively, are needed to be effective in the middle cerebral artery (2.5-mm diameter) or in the internal carotid artery (4 mm) in an average patient (9). The solo use of large-bore catheters resulted in better recanalization outcomes and significantly reduced distal emboli for internal carotid artery and MCA occlusions compared with all other devices and combinations *in vitro* (8). With the goal of TICI 3 in 10 min, Zoom Aspiration Catheters are designed for optimized clot engagement, effortless navigation and uncompromised structural integrity.

**Figure 4:**
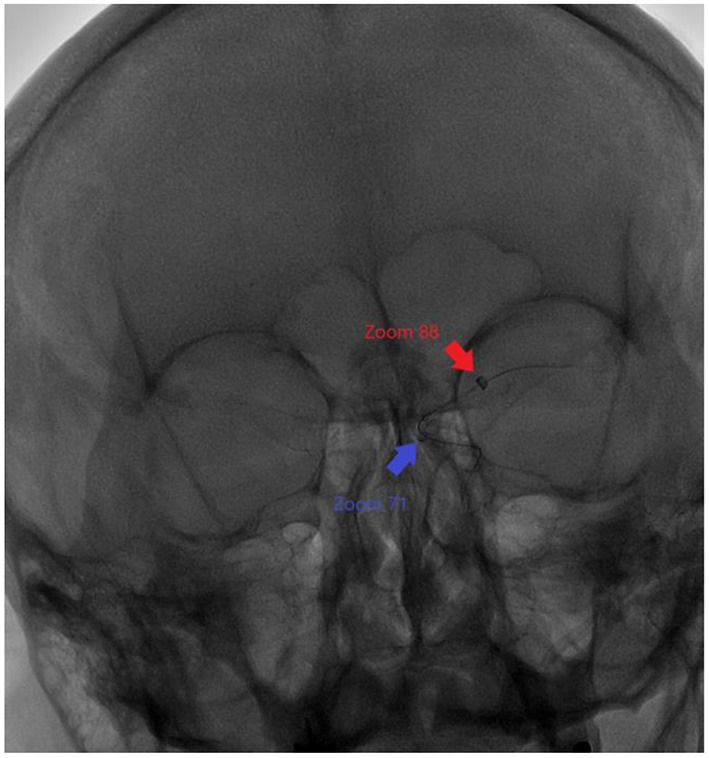
Relative positions of Zoom 88 and Zoom 71 catheters to each other during the mechanical thrombectomy procedure.

Here, the Zoom 088 catheter was advanced over the Zoom 71/velocity construct to prevent injury to arteries and ensure a smooth transition to the site of interest as pictured in Figure 4. While designed to get as close to the face of the clot as possible, it was advanced further past the clot into distal segments of the MCA in order to aspirate secondary emboli coming from disturbance of the primary clot. Given the large bore catheter size, it is important to be careful with how quickly it is advanced and how distal it travels when the inner catheters that facilitate its transition are removed as the vessels in the brain are small and therefore easily prone to damage. Sometimes the stored potential energy from the multiple loops in the head and neck can allow the large-bore catheters to travel further into the brain than warranted, leading to complications, such as what happened in this case. Based on this information, it can possibly be surmised that there may have been a technical error in the way the catheter had been handled during the case, but not because of any defects of the catheter itself. On a different note, it can also be argued that the unique shape of the beveled tip of the catheter may cause complications, such as dissections, but it has been shown by Vargas et al. that patients who underwent mechanical thrombectomy with the beveled tip catheter have a higher proportion of TICI 2C or better and a significantly lower mRS score on discharge and at 90 days, though this does not rule out the possibility of dissection (10). There is also the likelihood of introducing infections into the cerebrovascular system or damaging the vasculature. Additionally, there are the issues of limited access to trained neurointerventionalists, technical difficulty with the navigating wire in delicate intracranial vessels, trauma to vessels, distal embolization, vessel dissection and vasospasm leading to worsening of stroke (11). The need for greater efficacy and efficiency still remains and larger prospective studies are warranted (6). Despite the drawbacks, both small- and large-scale studies have consistently shown how beneficial large-bore catheters are.

According to one study, five patients (ages 50–85 years; baseline NIHSS 17–23) were treated. The 0.088”catheters were used as the primary tool for contact aspiration in two patients (distal basilar artery and proximal MCA occlusions) with complete thrombus ingestion (eTICI3) during the first pass. Out of the five patients, the 0.088”catheter was used for flow control in two of them. As the first patient presented with an M2 occlusion, the catheter was placed in the distal M1 segment and treated with a combination of a stent-retriever and 0.070”aspiration catheter; the second patient came in with a distal M1 occlusion, so the catheter was placed in the proximal M1 segment and treated with a 0.071”aspiration catheter. In the end, both patients had an eTICI3 reperfusion score. Additionally, it should be noted that the fifth patient in the same study had the 0.088”catheter navigated into the cavernous ICA to support 0.071”aspiration catheter treatment of an M2 occlusion resulting in eTCI2B67 reperfusion. Procedural duration ranged between 14 and 33 min. There were no adverse events, further substantiating the use of large-bore catheters during mechanical thrombectomies (6).

In another study by Tonetti et al. (5) 464 cases of large-vessel thrombectomies using multiple large-bore catheters were scrutinized, 180 of which were done *via* MAT using various large-bore catheters. The findings of this study concluded that the choice of the catheter had no influence on clinical outcomes, first-pass aspiration, or final reperfusion (5). Out of the four catheters this study looked at, it was found the first-pass success rate did not differ significant between the Sofia, CAT6, 0.072-inch Navien, and ACE68, falling between 36 and 50% with a *p* = 0.67 (5). Additionally, TICI reperfusion scores of 2B or higher was achieved in 94% of cases overall, *p* = 0.70. Lastly, there was no significant difference between 90-day good outcome and 90-day mortality in patients treated with different catheters, suggesting that large-bore catheters in general can prove quite useful in mechanical thrombectomy procedures.

## Conclusion

Strokes can be treated both medically and by interventional means. As far as the interventional means are concerned, mechanical thrombectomy appears to be an increasingly popular option for patients who qualify. On the other hand, it should be noted that there are disadvantages, such as clot reformation or instrumental error. In our case, the patient passed away on the third post-operative day secondary to non-hemorrhagic left hemispheric infarction involving both the middle cerebral and posterior communicating arteries from an overshot catheter. While unfortunate, large-bore catheters have still proven to be highly successful in the retrieval of clots. Both small-scale and large-scale have demonstrated their usefulness, and while more studies are always encouraged, so far, the benefits outweigh the risks and the safety and efficacy of their use is fruitful.

## Data availability statement

The datasets presented in this article are not readily available because of ethical and privacy restrictions. Requests to access the datasets should be directed to the corresponding author.

## Ethics statement

Ethical review and approval was not required for the study on human participants in accordance with the local legislation and institutional requirements. Written informed consent for participation was not required for this study in accordance with the national legislation and the institutional requirements. Written informed consent was not obtained from the individual(s) for the publication of any potentially identifiable images or data included in this article.

## Author contributions

MB and CB wrote and edited the manuscript. HS and BJ edited the manuscript. All authors contributed to the article and approved the submitted version.
