# Supplementary material for: Case report: Utilization and efficacy of large-bore catheters in mechanical thrombectomies
Source: Front Neurol. 2023 Jan 10;13:1035959. doi: 10.3389/fneur.2022.1035959 (PMC9871883; doi:10.3389/fneur.2022.1035959)
Supplement: Supplementary file 1 [file Table_1.DOCX]

Table 1: Neurointerventional Radiology Aspiration Catheter Sizes and Lengths

| **Name of Catheter** | **Vendor** | **Inner Diameter (inches)** | **Outer Diameter (inches)** | **Length (centimeters)** |
| --- | --- | --- | --- | --- |
| Zoom 088 | Imperative Care | .088 | .110 | 110 |
| Zoom 71 Reperfusion | Imperative Care | .071 | .083 | 137 |
| AXS Vecta 71 | Stryker (Neuro) | .071 | .085 | 132 |
| AXS Vecta 74 | Stryker (Neuro) | .074 | .087 | 132 |
| React 71 | MedTronic | .071 | .085 | 132 |
| React 68 | MedTronic | .074 | .087 | 132 |
| Hi-Point 088 | Route 92 Medical | .088 | .101 | 65/143* |
| Red 62 | Penumbra | .062 | .076 | 138 |

*Catheter Length/Total Length
